# Genomic analysis of the polyamine biosynthesis pathway in duckweed *Spirodela polyrhiza* L.: presence of the arginine decarboxylase pathway, absence of the ornithine decarboxylase pathway, and response to abiotic stresses

**DOI:** 10.1007/s00425-021-03755-5

**Published:** 2021-10-25

**Authors:** Rakesh K. Upadhyay, Jonathan Shao, Autar K. Mattoo

**Affiliations:** 1 Sustainable Agricultural Systems Laboratory, United States Department of Agriculture, Agricultural Research Service, Henry A. Wallace Beltsville Agricultural Research Center, Beltsville, MD 20705-2350 USA; 2 Bioinformatics-North East Area Office, United States Department of Agriculture, Agricultural Research Service, Henry A. Wallace Beltsville Agricultural Research Center, Beltsville, MD 20705-2350 USA

**Keywords:** *Spirodela polyrhiza*, Duckweed, Polyamines, Putrescine, Spermidine, Spermine, Methyl Jasmonate, Salinity

## Abstract

**Main conclusion:**

Identification of the polyamine biosynthetic pathway genes in duckweed *S. polyrhiza* reveals presence of prokaryotic as well as land plant-type ADC pathway but absence of ODC encoding genes. Their differential gene expression and transcript abundance is shown modulated by exogenous methyl jasmonate, salinity, and acidic pH.

**Abstract:**

Genetic components encoding for polyamine (PA) biosynthetic pathway are known in several land plant species; however, little is known about them in aquatic plants. We utilized recently sequenced three duckweed (*Spirodela polyrhiza)* genome assemblies to map PA biosynthetic pathway genes in *S. polyrhiza*. PA biosynthesis in most higher plants except for *Arabidopsis* involves two pathways, via arginine decarboxylase (ADC) and ornithine decarboxylase (ODC). ADC-mediated PA biosynthetic pathway genes, namely, one *arginase* (*SpARG1*), two *arginine decarboxylases* (*SpADC1*, *SpADC2*), one *agmatine iminohydrolase*/*deiminase* (*SpAIH*), one *N-carbamoyl putrescine amidase (SpCPA)*, three *S-adenosylmethionine decarboxylases* (*SpSAMDc1, 2, 3*), one *spermidine synthase* (*SpSPDS1)* and one *spermine synthase* (*SpSPMS1*) in *S. polyrhiza* genome were identified here. However, no locus was found for ODC pathway genes in this duckweed. Hidden Markov Model protein domain analysis established that *SpADC1* is a prokaryotic/biodegradative type ADC and its molecular phylogenic classification fell in a separate prokaryotic origin ADC clade with *SpADC2* as a biosynthetic type of arginine decarboxylase. However, *thermospermine synthase* (*t-SPMS)/Aculis5* genes were not found present. Instead, one of the annotated *SPDS* may also function as *SPMS,* since it was found associated with the *SPMS* phylogenetic clade along with known *SPMS* genes. Moreover, we demonstrate that *S. polyrhiza* PA biosynthetic gene transcripts are differentially expressed in response to unfavorable conditions, such as exogenously added salt, methyl jasmonate, or acidic pH environment as well as in extreme temperature regimes. Thus, *S. polyrhiza* genome encodes for complete polyamine biosynthesis pathway and the genes are transcriptionally active in response to changing environmental conditions suggesting an important role of polyamines in this aquatic plant.

**Supplementary Information:**

The online version contains supplementary material available at 10.1007/s00425-021-03755-5.

## Introduction

The ubiquitous polyamines (PAs) are small aliphatic biogenic amines that have been shown to impact many aspects of biological processes in many genera including plants (Kushad et al. 1988; Carbonell and Navarro 1989; Flores 1991; Cohen 1998; Mehta et al. 2002; Bregoli et al. 2002; Kusano et al. 2008; Nambeesan et al. 2010, 2019; Alcázar et al. 2010). In plants, the most abundant PAs include putrescine (Put), spermidine (Spd), and spermine (Spm), while others such as cadaverine, thermospermine, norspermidine, and norspermine are relatively less abundant (Cohen 1998). Early works have demonstrated that PAs inhibit biosynthesis of the plant hormone ethylene in higher plants (Apelbaum et al. 1981; Ben-Arie et al. 1982) and shunt label from 3,4-[^14^C]methionine into SPD in aged orange peel discs (Even-Chen et al. 1982). Subsequently, their role(s) in plant development, morphogenesis, senescence, fruit set, fruit ripening, anabolic and nitrogen–carbon interactions were unearthed (Biasi et al. 1988; Rastogi and Davies 1990; Shiozaki et al. 2000; Mehta et al. 2002; Bregoli et al. 2002; Malik and Singh 2004; Tassoni et al. 2004; Liu et al. 2006; Ziosi et al. 2006; Mattoo et al. 2006; Gomez-Jimenez et al. 2010; Nambeesan et al. 2019).Transgenic approaches demonstrated different plant responses to diamine Put versus Spd and Spm (Mattoo et al. 2010) as well as the presence of a nexus between Spm and floral organ identity and fruit set (Nambeesan et al. 2019).

PA content and function are largely regulated by changes in their synthesis and breakdown based on PA homeostasis (Mattoo et al. 2015; Handa et al. 2018). Put synthesis is initiated from arginine catalyzed by arginine decarboxylase (ADC) or/and from ornithine catalyzed by ornithine decarboxylase (ODC), respectively. Conversion of Put to Spd is catalyzed by Spd synthase (SPDS) and, in turn, Spm/T-Spm biosynthesis of Spm and T-Spm are mediated by spermine synthase (SPMS) and thermospermine synthase (ACL5), respectively. The amino propyl groups used for the synthesis of SPD and SPM are generated by S-adenosylmethionine decarboxylase (SAMDc) during the conversion of S-adenosylmethionine (SAM) to decarboxylated S-adenosylmethionine (SAMdc). Put catabolism is primarily mediated by diamine oxidases (CuAOs), while the terminal catabolism or back conversion of triamine SPD and tetramine SPM is modulated by polyamine oxidases (PAOs) (Cona et al. 2006; Kusano et al. 2008; Planas-Portell et al. 2013; Kim et al. 2014; Liu et al. 2015).

Aquatic higher plants called duckweeds (Lemnaceae) with reduced anatomies have re-emerged as models for higher plants particularly after genomes of several duckweed species were recently sequenced (An et al. 2018, 2019; Hoang et al. 2018, 2020; Acosta et al. 2021). Previous research has highlighted duckweeds as a model for photosynthesis research which uncovered the dynamics of the photosystem II D1 protein (Mattoo et al. 1981, 1984, 1989; Mattoo and Edelman 1987) as well as important aspects connected with ecotoxicological and phytoremediation studies (Ziegler et al. 2016). Duckweed as a supplement for food with higher nutritional value for human consumption and biomanufacturing has attracted a lot of attention (Hillman 1961; Rusoff et al. 1980; Stomp 2005; Wang et al. 2014; Van Hoeck et al. 2015; Appenroth et al. 2015; Edelman and Colt 2016).

Little is known about the role of PAs in the growth, development, and stress tolerance in duckweed. Here, a comprehensive bioinformatics analysis was carried out to map the PA biosynthetic pathway-related genes in duckweed utilizing the genomic database hosted at phytozome (*S. polyrhiza7498v2)* (Wang et al. 2014)*,* and the two new genome assemblies (*S. polyrhiza7498v3*) (An et al. 2019) and (*S. polyrhiza9505v3*) (Michael et al. 2017). We have identified the candidate duckweed genes involved in PA biosynthesis and analyzed their gene structure, phylogenetic relationships, expression profiles during growth, and their patterns in response to exogenously applied phytohormone methyl jasmonate (MeJA) as well as salinity stress and acidic pH. We also present impact of four abiotic stresses on the modulating transcript accumulation of PA pathway genes in duckweed. The functional relevance of PA metabolic pathway unraveled in duckweed is discussed.

## Materials and methods

### Retrieving PA biosynthesis pathway encoding genes sequences from *Spirodela polyrhiza* genome

Genome sequence from *Spirodela polyrhiza* strain 7498v2 hosted at phytozome database (http://www.phytozome.net/) (Wang et al. 2014) and another assembly from NCBI, *Spirodela polyrhiza* strain 7498v3 (An et al. 2019), were utilized to retrieve putative duckweed (*S. polyrhiza*) polyamine metabolic pathway gene transcripts and protein sequences. Multiple bioinformatics approaches were employed to identify and characterize potential genes in *S. polyrhiza* as previously described (Upadhyay and Mattoo 2018). PA biosynthetic pathway encoding sequences for arginase/agmatinase (ARG), arginine decarboxylase (ADC), ornithine decarboxylase (ODC), *N*-carbamoylputrescine amidase (CPA), agmatine iminohydroxylase (AIH) or agmatine deiminase, *S*-adenosylmethionine decarboxylase (SAMDc) spermidine synthase (SPDS), and spermine synthase (SPMS) from Arabidopsis (Majumdar et al. 2017), tomato (Liu et al. 2018; Upadhyay et al. 2020b) and rice (http://www.phytozome.net/) were downloaded and used as a query against *S. polyrhiza* genome to search for similar sequences. BLASTp search (*E* value, 10^−5^) was used to search for similar protein sequences in *S. polyrhiza* genome. Putative PA pathway protein sequences of *S. polyrhiza* were analyzed using Hidden Markov Model (HMM) analysis for the presence of a typical HMM domain representing the corresponding protein class in a HMMER search (https://www.ebi.ac.uk/Tools/hmmer/) (Finn et al. 2011; Upadhyay and Mattoo 2018). For accuracy these sequences were also cross verified with another genome assembly of *S. polyrhiza* strain 7498v3 (NCBI accession: GenBank assembly accession: GCA_008360905.1] (An et al. 2019). In addition, a cross-verification for all PA genes for their genomic locations was carried out with another genome assembly, *S. polyrhiza* 9509 v3 (GenBank assembly accession: GCA_900492545.1) (Michael et al. 2017). Gene nomenclature was based on their occurrence on pseudomolecules. The predicted molecular weight and isoelectric point (pI) for each protein were obtained using tools available at the ExPASy bioinformatics resource portal (https://www.expasy.org).

### Gene prediction and annotation of five incomplete genes of *S. polyrhiza* genome

Based on above searches, putative gene sequences [(SpADC1 (Spipo9G0068800), SpCPA (Spipo0G0005000), SpSAMDc1 (Spipo1G0013800), SpSAMDc2 (Spipo5G0023000), SpSAMDc3 (Spipo9G0049500), were downloaded from phytozyme (https://phytozome.jgi.doe.gov/pz/portal.html). These gene models were incomplete likely due to sequencing error(s) or incomplete contig sequences. Therefore, the sequences were blasted (BLASTN) against the unannotated duckweed (*S. polyrhiza*) genome Genbank Assembly accession GCA_008360905.1, WGS project SWLF01.1 (https://www.ncbi.nlm.nih.gov/assembly/GCA_008360905.1/) to retrieve the positions of the genes in the contigs of the Duckweed (*S. polyrhiza*) genome (An et al. 2019). Fragments from sequence data were extracted from the duckweed (*S. polyrhiza*) genome and manual curation was carried out using various gene finders and alignments. Gene calls were made in the Augustus gene finder using *Oryza brachyantha*, *Zea mays* and *Triticum aestivum* as model (http://bioinf.uni-greifswald.de/augustus/) (Stanke et al. 2004), and in the FGENESH program (http://www.softberry.com/) (Solovyev et al. 2006) using *Zea Mays* and *Oryza sativa* var. Indica as the model. A Perl script was also written to extract the relevant intron and exons from the output to build gene models. Alignments were done in the Megalign program from the DNASTAR software package (DNASTAR. Madison, WI.). Conserved domains in genes/proteins obtained from gene models, were verified in HMMER (https://www.ebi.ac.uk/Tools/hmmer/) (Potter et al. 2018).

### Molecular phylogenetic analysis of *S. polyrhiza* PA biosythetic pathway protein sequences

Sequences of genes from model plants, namely, *Arabidopsis*, tomato, and rice were selected for analyzing *ADC*, *ODC*, *AIH*, *CPA*, *SAMDc* and *SPDS* genes. In addition, BLASTp search using 10 pathway sequences yielded many sequences from NCBI. Due to a large number of sequences, a cutoff of 50% sequence identity was taken into consideration for pooling the sequences for reconstruction of the phylogeny. Sequences from lotus (*Nelumbo nucifera*) and seagrass (*Zostera marina*) were chosen to establish the relationship with complete aquatic nature, partial aquatic, and costal habitat plants. Multiple sequence alignments were performed using the MUSCLE program (http://www.ebi.ac.uk/Tools/msa) (Edgar 2004). A phylogenetic tree was constructed using the maximum likelihood method with Poisson correction and 1000 bootstrap values (Zuckerkandl and Pauling 1965; Felsenstein 1985), while the tree was displayed using the MEGA7 program (Kumar et al. 2016). Sequence identifiers used to reconstruct the phylogeny are listed in Supplementary Table 1.

### Identification of conserved motifs, subcellular localizations, gene structure and gene duplication analyses

PA pathway sequences were submitted to the Hidden Markov Model (HMM) analysis database to find respective PFAM domains for ARG, ADC, ODC, CPA, AIH or agmatine deiminase, SAMDc, SPDS and SPMS, respectively. Identification of signal peptides and protein localization studies were carried out using Phobius (https://phobius.sbc.su.se/) and TargetP2.0 (http://www.cbs.dtu.dk/services/TargetP/) analyses. For gene structure analysis, genomic DNA and coding DNA sequences corresponding to each identified gene were analyzed for intron–exon and intron phase distribution patterns (http://gsds.cbi.pku.edu.cn/). Similarity index for nucleotides and amino acids were calculated using clustal omega and MUSCLE, and displayed using clustal 2.1 (https://www.ebi.ac.uk/Tools). More than 90% sequence similarity among genes was considered as segmental duplication (Sharp et al. 2005). Tandemly duplicated gene pairs were identified as described previously (Yang et al. 2008; Upadhyay and Mattoo 2018).

### Plant material, growth conditions, MeJA or salinity treatments

Two duckweed strains (*S. polyrhiza,* 7498 and 7003) were grown at room temperature (22 ± 2 °C) under white light (50–80 μmol/s^2^) as previously described (Upadhyay et al. 2020a). Three growth periods (14 ± 1 day, 21 ± 1 day and 28 ± 1 day) were chosen as previously described (Mattoo et al. 1992). All experiments were carried out in triplicate. Samples were taken at indicated timepoints, washed thrice with distilled water, frozen in liquid nitrogen and stored at − 70 °C until used. Transcript abundance of *S. polyrhiza* 7498 PA biosynthetic pathway genes in response to MeJA was carried out as previously described (Upadhyay et al. 2020a). MeJA (Sigma, 95%) was diluted 1:10 with 95% ethanol, followed by a further dilution with sterile MilliQ water containing 0.1% Triton X-100, to a final concentration of 10 μM MeJA. Two batches of 14-day grown duckweeds (each batch consisting of 200–300 fronds) were collected from nutrient solution and washed twice with previously autoclaved distilled water. MeJA solution was applied to plants for 12 h with one batch kept without MeJA as control. Samples were collected in triplicate at 0, 1, 3, 6 and 12 h post-treatment. A minimum of 10–15 fronds were analyzed for each timepoint. Salinity stress involved treatment of 14-day-old *S. polyrhiza* 7498 plants with 200 mM salinity (Upadhyay et al. 2020a). In total 100 fronds were transferred into flasks and a solution of 200 mM NaCl was added. Control plants were not exposed to salinity. Samples were collected in triplicate at 0, 1, 3, 6 and 12 h post-treatment. A minimum of 10–15 fronds at each timepoint were sampled, frozen in liquid nitrogen and stored at − 70 °C until used.

### Growth of *S. polyrhiza* at acidic pH conditions

To determine if duckweed PA pathway genes respond to an acid environment, we grew 14-day-old duckweed for 12 h at three physiological pH conditions, pH 5.8 (control), pH 3.0 and pH 1.0. A total of 100 fronds in each flask were tested at these pH conditions. Samples were collected in triplicate at 0, 1, 3, 6 and 12 h post-treatment. A minimum of 10–15 fronds were analyzed at each timepoint. The collected samples were frozen in liquid nitrogen and stored at − 70 °C until used.

### Temperature stress (heat/cold) treatments to Duckweed (*S. polyrhiza *L.*7*498)

Heat stress treatment was carried out as described earlier with modifications (Upadhyay et al. 2019). Briefly, heat treatment included exposure of 14-day grown duckweed plants. The flasks were placed at a temperature maintaining growth chamber at 42 °C for 12 h. Time of day/control plants were also sampled at the same time as the control temperature. 10–15 fronds were collected from the heat-treated plants at 0, 1, 3, 6 and 12 h (three biological replicates). Harvested samples were immediately used for total RNA isolation. Low temperature or cold stress treatment was carried out as described earlier (Upadhyay et al. 2019). Briefly, cold treatment was given to 14-day grown duckweed plants by placing whole flasks in a walk-in cold room (4 °C) with 200 μmol/m2 light photoperiod. Experiment was started at 8.00 am EST (Eastern Standard Time). Leaf samples were collected thereafter at 0, 1, 3, 6 and 12 h. The remaining process was the same as above for heat stress.

### Total RNA extraction, cDNA preparation and quantitative real time PCR (qRT-PCR)

Total RNA was extracted from 100 mg of each sample using the plant RNeasy kit according to manufacturer’s instructions (Qiagen). RNA samples with an A_*260/280*_ ratio of 1.8–2.0 were then electrophoresed on agarose gels to ensure the presence of intact rRNA bands. Methods for cDNA synthesis and qRT-PCR were essentially as described previously (Bustin et al. 2009; Upadhyay et al. 2019). An iScript Advanced cDNA synthesis kit and SsoAdvanced universal SYBR green super mix reagents were used for qRT-PCR (Bio-Rad, Hercules, California, USA). The CFX-96 real-time PCR detection system was used for gene expression quantification (Bio-Rad, Hercules, California, USA). Relative gene expression was quantified using the 2^−∆∆CT^ method (Livak and Schmittgen 2001). *S. polyrhiza* actin (Spipo17G0011400) and 18S rRNA (Spipo23G0000600) genes were used as standard housekeeping genes to normalize the expression of target genes (Upadhyay et al. 2020a). qRT-PCR data represent the average ± standard deviation of a minimum of three independent biological replicates for each gene. Primer 3 tool/NCBI Primer-Blast tool was used for primer designing. Each primer sequence was tested with a blast search in the genome of *S. polyrhiza* 7498 for specific hits and for its specificity to yield a single amplicon on 1.2% agarose gel. Primers used in this study are listed in Supplementary Table 2.

### Gene bank and sequencing data information

Duckweed (*S. polyrhiza*7498v1) genome assembly hosed at Phytozome was used for initial queries, while final results were obtained using improved *S. polyrhiza*7498v3 genome assembly [gene bank assembly accession: GCA_008360905.1 (An et al. 2019)]. *S. polyrhiza* 9509 v3 genome raw assembly [gene bank assembly accession: GCA_900492545.1) (Michael et al. 2017)] was used to cross check the sequences. Protein sequences from various other plants, used in phylogenetic tree to prepare Figs. 3 and 4 are listed in Supplementary Tables 1, 2, and 3.

### Data analysis

The GraphPad (version8.0) suite was used for statistical analysis. ANOVA was performed for significant differences in LOX gene expression. For the MeJA, salinity treatments and temperature, significant differences were calculated against non-treated control samples at each timepoint, and categorized at *P < 0.05, **P < 0.01 and ****P < 0.0001 for each analysis (Upadhyay et al. 2019). Significant differences and *P* value are shown in Supplementary Table 3.

## Results

### Identification of PA biosynthetic pathway genes in the greater duckweed *S. polyrhiza*

A Hidden Morkov Model (HMM) assisted protein homology search was carried out with available PA metabolic pathway proteins from Arabidopsis, rice and tomato as described earlier (Upadhyay and Mattoo 2018). Sequence similarities led us to the identification of 10 *bona fide* PA biosynthetic pathway genes in the *S. polyrhiza* genome (Table 1). These include one arginase *SpARG1(*Spipo17G0020700), two arginine decarboxylases *SpADC1* (Spipo9G0068800) and *SpADC2* (Spipo16G0022600), and three S-adenosylmethionine decarboxylases [encoded by three genes *SpSAMDc1* (Spipo1G0013800), *SpSAMDc2* (Spipo5G0023000) and *SpSAMDc3* (Spipo9G0049500)]. Two loci annotated as spermidine synthases were present in the released assembly hosted at phytozome. Based on BLASTp searches in NCBI, out of the two one is spermidine synthase gene *SpSPDS1(*Spipo12G0011300*)* and the other spermine synthase gene *SpSPMS1* (Spipo26G0016400). In this search, no thermospermine synthase-like (tSPMS/ACULIS5-like) gene was found in the *S. polyrhiza* genome.

**Table 1:** PA biosynthetic pathway enzymes encoding genes found in Duckweed (*S. polyrhiza*) genome

Pathway	Name	Sequence ID	Location coordinates	Genomic	Transcript	CDS	Protein	pI	Mol Wt
PUT biosynthesis	SpARG1	Spipo17G0020700	pseudo17:1,702,637–1,705,311	2675	1014	1014	337^**#**^	5.81	36.79
	SpADC1	Spipo9G0068800	pseudo9:4,817,792–4,819,333 (−)	1689	1689	1689	562*	5.54	59.32
	SpADC2	Spipo16G0022600	pseudo16:1,712,924–1,715,107 (−)	2184	2184	2184	727^**#**^	5.25	77.41
	SpCPA	Spipo0G0005000	pseudo0: 275,702–277,444 ( +)	1743	930	930	309*	6.16	34.60
	SpAIH	Spipo25G0016900	pseudo25:1,812,627–1,818,892 (−)	6266	1149	1149	382^**#**^	4.94	42.73
SPD/SPM biosynthesis	SpSAMDc1	Spipo1G0013800	pseudo1:888,984–890,081 (−)	1158	1158	1158	385*	5.16	42.24
	SpSAMDc2	Spipo5G0023000	pseudo5:1,673,482–1,674,546 (−)	1032	1032	1032	343*	5.47	37.03
	SpSAMDc3	Spipo9G0049500	pseudo9:3,212,539–3,213,663 (−)	1128	1128	1128	375*	5.22	40.76
	SpSPDS1	Spipo12G0011300	pseudo12:1,139,820–1,142,885 (-)	3066	882	882	293^**#**^	4.89	32.17
	SpSPMS1	Spipo26G0016400	pseudo26:1,056,440–1,060,492 (+)	4053	1035	1035	344^**#**^	5.06	37.78

*These protein encoding genes were truanted due to sequencing error at phytozome hosted genome assembly (*S. polyrhiza*7498v2 (NCBI assembly#GCA_000504445.1)]. The new assembly from NCBI [*S. polyrhiza*7498v3 (NCBI assembly# GCA_008360905.1)] was used to complete these sequences

^**#**^These protein encoding genes were having similar size of encoded proteins from both assemblies

Genomic loci for ornithine decarboxylase (ODC) encoding gene(s) across three *S. polyrhiza* genome assemblies, namely, Sp7498v2 (Phytozome v12; GenBank assembly accession: GCA_000504445.1), Sp7498v3 (GenBank assembly accession: GCA_008360905.1) and Sp9509v2 (GenBank assembly accession: GCA_900492545.1) were not found. Diaminopimelate decarboxylase *SpDAPDC1* (Spipo8G0054200) which shares close similarity to ADC/ODC-like sequences was identified via shared HMM profile but its direct participation in PA biosynthesis is not known. Table 1 shows the genomic locations, transcripts, coding fragment and encoded protein sizes along with their molecular weights and isoelectric points (pI) of ten identified PA biosynthetic pathway encoding genes.

### Gene structure of PA pathway encoding genes

**Fig. 1:**
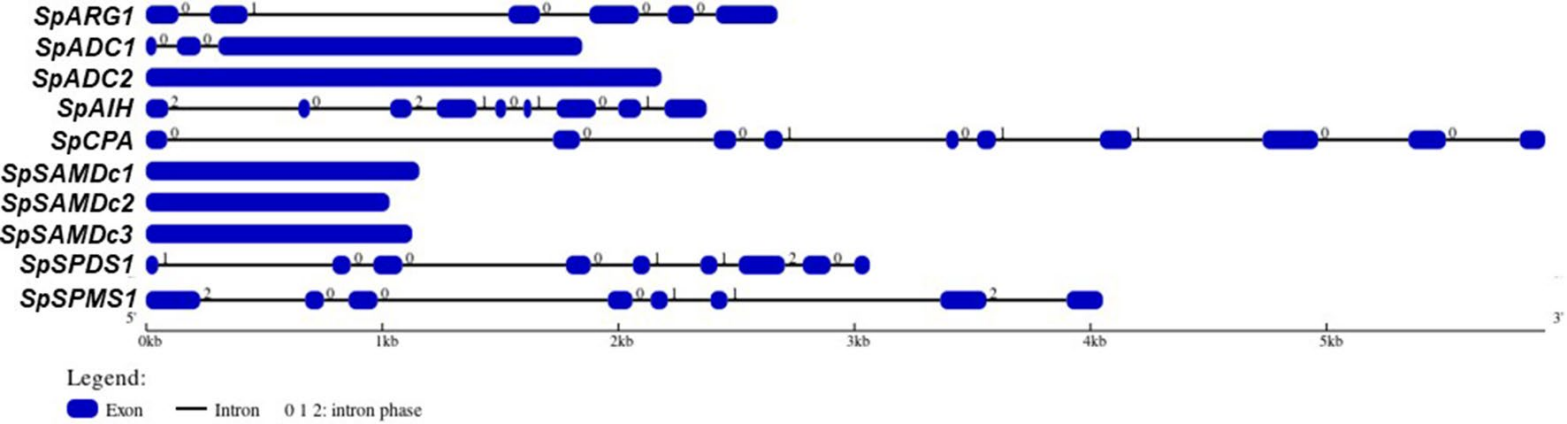
Gene structure of PA biosynthetic pathway encoding genes. Genomic DNA and coding DNA sequences corresponding to each identified gene were analyzed for intron–exon and intron phase distribution patterns (http://gsds.cbi.pku.edu.cn/). Intron–exon organization of the PA biosynthetic pathway genes indicated that some of the genes are intron-less, while others contain varying number of introns

Intron–exon organization of the PA biosynthetic pathway genes indicated that some of the genes are intron-less, while others contain a varying number of introns. Thus, *SpARG1* contains 6 exons separated by 7 intergenic regions, *SpADC1* contains 3 exons but *SpADC2* is intron-less, while *SpAIH* contains 9 exons and *SpCPA* 10 exons; three *SAMDc* genes are intron-less, while *SpSPDS1* possesses 9 and *SpSPMS1* 8 exons, respectively. Between *SpSPDS1* and *SpSPMS1* five exon/intron phases were found to be similar and may indicate their origin from a common ancestral gene (Fig. 1).

### Analysis of protein domains in PA biosynthetic pathway proteins

Domain annotation revealed that *SpARG1* possesses the HMM profile (PF00491) common to known arginases in *Arabidopsis*, tomato, and rice. Among the two arginine decarboxylases, *SpADC1* possesses two HMM domains, PF 01276 (Orn/Lys/Arg decarboxylase major domain) and PF 03711 (Orn/Lys/Arg decarboxylase C-terminal domain). These are mainly found in the known ODC/LDC/ADC group III pyridoxal phosphate-dependent decarboxylases (Table 2). On the other hand, *SpADC2* belongs to group IV pyridoxal-dependent decarboxylases common to ADC/ODC proteins in higher plants. *SpADC2* (Spipo16G0022600) possesses HMM profile of PF02784 with 20 aa long N-terminus signal peptide (Table 2). *SpCPA* (Spipo0G0005000) and *SpAIH* (Spipo25G0016900) contain HMM profiles of PF00795 and PF0471, respectively. Three S-adenosylmethionine protein genes, *SpSAMDc1* (Spipo1G0013800), *SpSAMDc2* (Spipo5G0023000) and *SpSAMDc3* (Spipo9G0049500) possess HHM profile (PF01536) similar to the land plant SAMDc proteins. Two SPDS/SPMS-like proteins, SpSPDS1 (Spipo12G0011300) and SpSPMS1 (Spipo26G0016400), contain the signature HMM profiles common for spermine/spermidine synthase domain (PF01564) and spermidine synthase tetramerization domain (PF17284) similar to SPDS/SPMS proteins of *Arabidopsis*, rice and tomato.

**Table 2:** Location of signature domain in *Spirodela polyrhiza* polyamine oxidase protein sequences

Gene (Sequence ID)	Size	Hidden Markov model domains (N → C)^1^
SpARG1 (Spipo17G0020700)	337	PF00491 (58–333)
SpADC1 (Spipo9G0068800)	562	PF01276 (74–383); PF03711 (478–551)
SpADC2 (Spipo16G0022600)	727	PF02784 (132–404)
SpCPA (Spipo0G0005000)	309	PF00795 (4–254)
SpAIH (Spipo25G0016900)	382	PF0471 (14–372)
SpSAMDc1 (Spipo1G0013800)	385	PF01536 (16–343)
SpSAMDc2 (Spipo5G0023000)	343	PF01536 (4–323)
SpSAMDc3 (Spipo9G0049500)	375	PF01536 (16–338)
SpSPDS1 (Spipo12G0011300)	293	PF17284 (7–61); PF01564 (64–252)
SpSPMS1 (Spipo26G0016400)	344	PF17284 (68–122); PF01564 (125–313)

^1^Hidden Markov Model analysis was done with HMMER as described previously (Upadhyay and Mattoo et al. 2018) and domains were located along with their positions in proteins sequences from N to C terminus of proteins

**Fig. 2:**
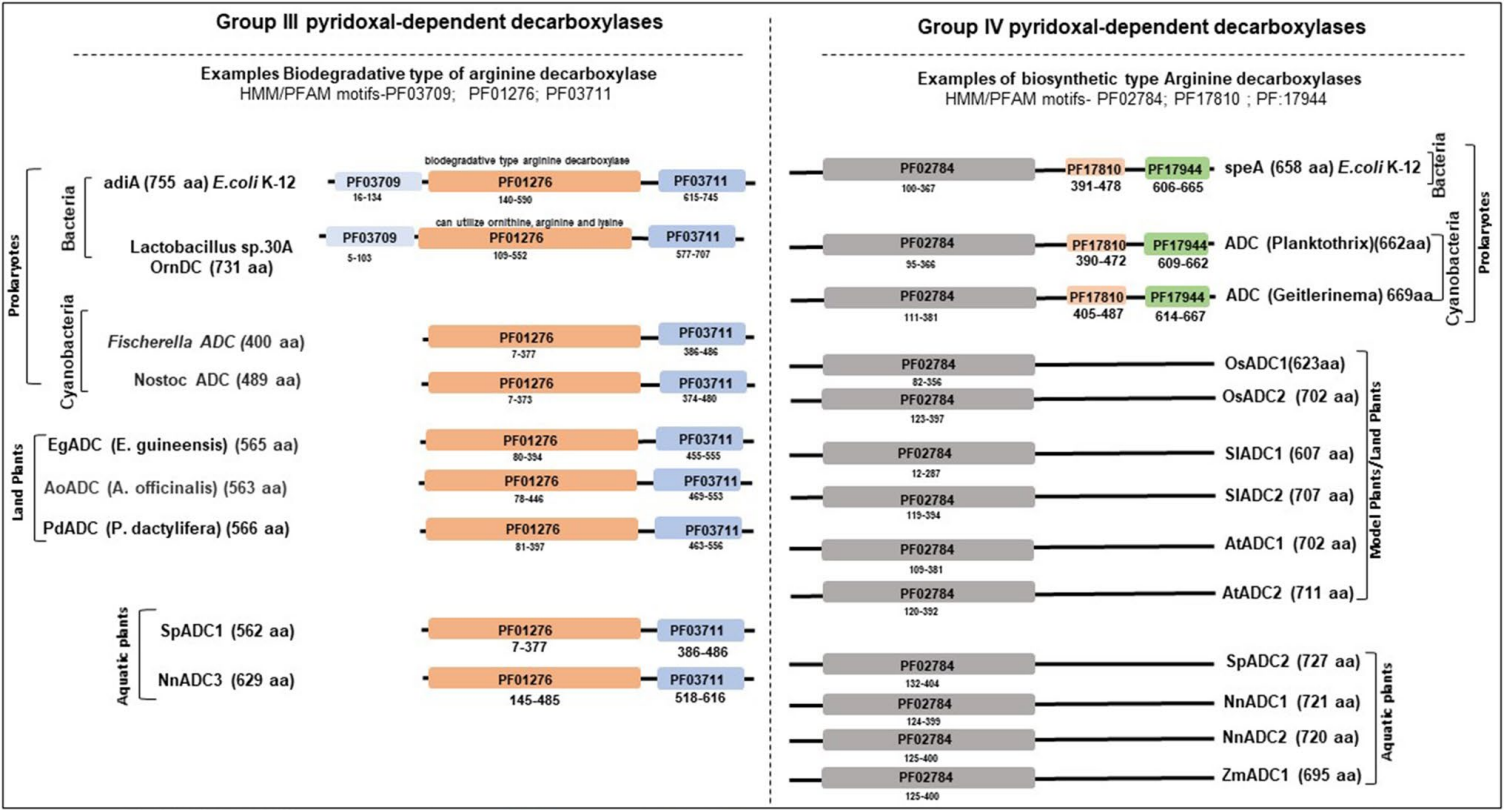
Comparison of two duckweed arginine decarboxylases with prokaryote and plant arginine decarboxylases. Two types of amino acid decarboxylase or pyridoxal-dependent decarboxylases participate in polyamine synthesis in prokaryotes, such as bacteria and plants—Groups III and IV. Group III comprises prokaryotic ornithine, lysine decarboxylase and the prokaryotic biodegradative type of arginine decarboxylase [Representative HMM profile or PFAM motifs are motifs-PF03709 (Orn/Lys/Arg decarboxylase N terminal domain); PF01276 (Orn/Lys/Arg decarboxylase major domain); PF03711(Orn/Lys/Arg decarboxylase C terminal domain). Group IV comprises eukaryotic ornithine and lysine decarboxylase and the prokaryotic biosynthetic type of arginine decarboxylase and diaminopimelate decarboxylase [Representative HMM profile or PFAM motifs are motifs: PF02784 (pyridoxal binding domain); PF17810 (arginine decarboxylase helical bundle domain); PF17944 **(**arginine decarboxylase C-terminal helical extension**)**]

Group III arginine decarboxylases (also known as biodegradative type of arginine decarboxylases) possess three types of signature pfam domains: PF03709 (Orn/Lys/Arg decarboxylase N terminal domain); PF01276 (Orn/Lys/Arg decarboxylase major domain); and PF03711(Orn/Lys/Arg decarboxylase C terminal domain), while group IV arginine decarboxylases (also known as biosynthetic type of arginine decarboxylases) possess PF02784 (pyridoxal binding domain); PF17810 (arginine decarboxylase helical bundle domain); and PF17944 **(**arginine decarboxylase C-terminal helical extension**)**]. The pfam domain analysis of known ADCs revealed that the bacteria and cyanobacteria have both kinds of arginine decarboxylases based on their protein domain architecture but model plants such as Arabidopsis, rice and tomato have only group IV or biosynthetic type arginine decarboxylases (Fig. 2). HMM-based protein domain comparison of both groups of arginine decarboxylases revealed that *S. polyrhiza* ADCs have likely originated from two different classes of arginine decarboxylases (Fig. 2).

### Molecular phylogeny reconstruction and evolution of *S. polyrhiza* PA metabolic pathway proteins

**Fig. 3:**
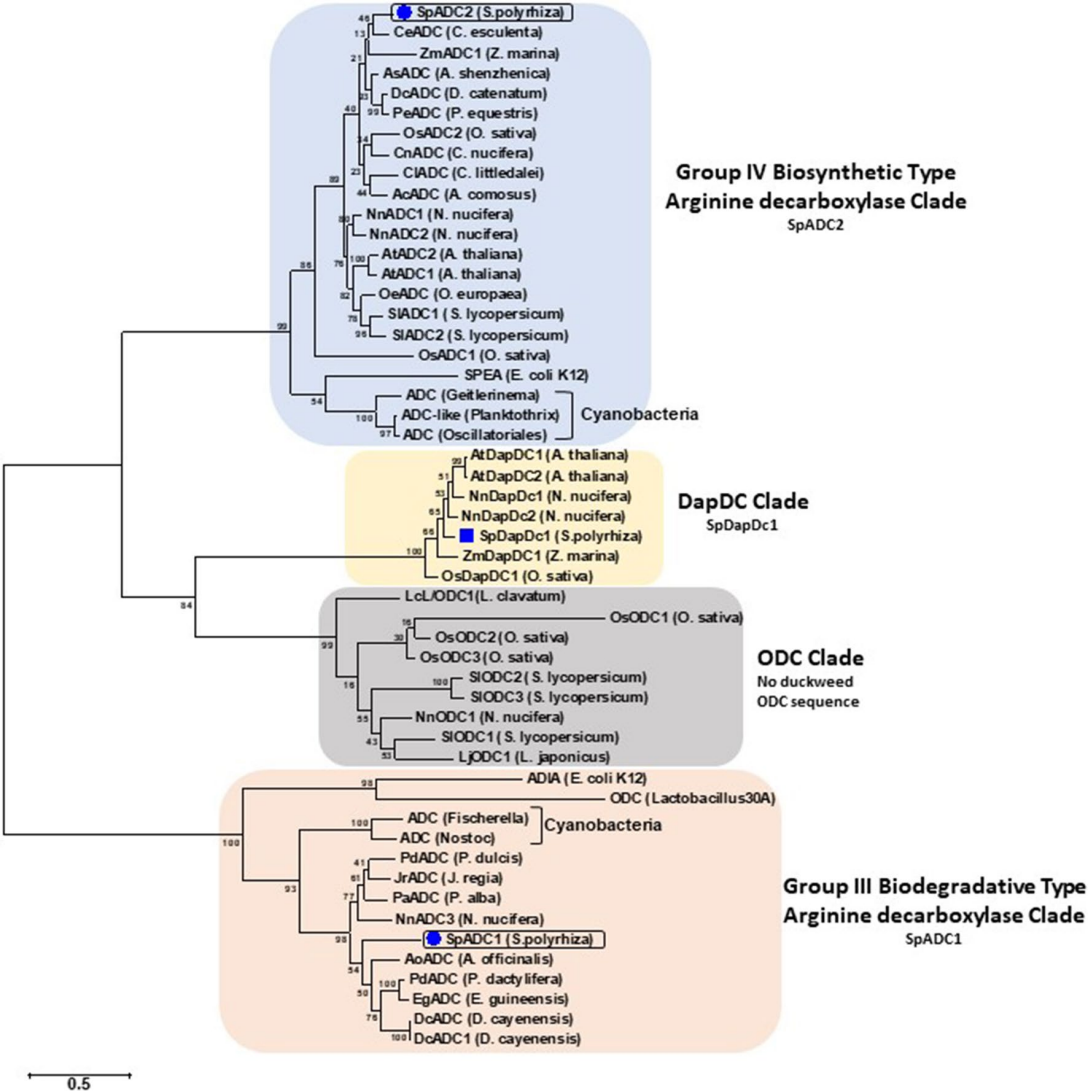
Molecular phylogenetic relatedness of arginine/ornithine/diaminopimelate decarboxylase encoding genes in duckweeds in comparison to known plant gene species. Molecular phylogenetic relationships of the *Spirodela polyrhiza* arginine/ornithine/diaminopimelate decarboxylase genes with similar sequences from known plants, was derived for establishing an evolutionary relationship. The evolutionary history was inferred using the maximum likelihood method based on the JTT matrix-based model. The bootstrap consensus tree inferred from 1000 replicates is taken to represent the evolutionary history of the taxa analyzed (Felsenstein 1985). The analysis involved 43 protein sequences from different plants. All positions with less than 95% site coverage were eliminated. Evolutionary analyses were conducted in MEGA7 (Kumar et al. 2016). The bootstrap values of the confidence levels are shown as percentages at branch nodes. ADC/ODC/DAPDC of different species fall into three separate clades: prokaryotic type land ADC clade, land plant type ADC clade, DAPDC clade and ODC clade

Gene sequences of ADC, ODC and diaminopimelate decarboxylases (DAPDC) from *Arabidopsis*, tomato and rice were chosen to establish phylogenetic relationship among protein sequences. In addition, two other genomes were screened, namely, seagrass/sea grass (*Zostera marina*) and lotus (*Nelumbo nucifera*), for ADC, ODC and DAPDC-like sequences. Phylogenetic tree drawn from these pooled sequences separated them into three distinct single clades—ADC clade, ODC clade and DAPDC clade (Fig. 3). In addition, ADC clade sequences were separable into two distinct sub-clades, one having all prokaryotic/biodegradative type ADC sequences and the other having all land plant type ADC sequences. In the phylogeny reconstruction, duckweed-related prokaryotic/biodegradative type *SpADC1* gene showed similarities with those plant ADC sequences that belong to aquatic/semi-aquatic or coastal region dwelling plants. The *SpDAPDC1* sequences fell into one clade so did all the ODC sequences (Table 3). For comparison, we also identified ODC-like sequences from lotus. Interestingly, aquatic plant genomes for duckweed (*S. polyrhiza*) and seagrass investigated in this study were found not to contain any genomic loci encoding for ODC-like sequences.

**Table 3:**
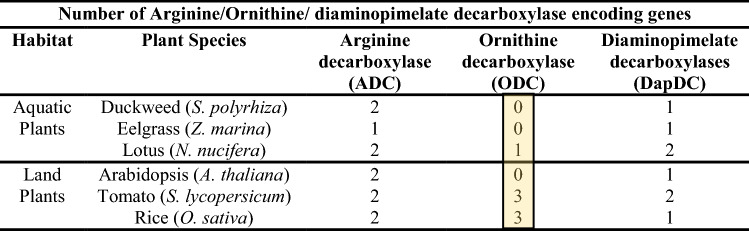
Comparative number of arginine/ornithine/diaminopimelate decarboxylase encoding genes in land plant vs aquatic plants

The seagrass and lotus amino acid decarboxylase sequences are not known. We modeled respective gene assemblies to identify these gene sequences for developing a comparative overview among land plant vs aquatic plant sequences

**Fig. 4:**
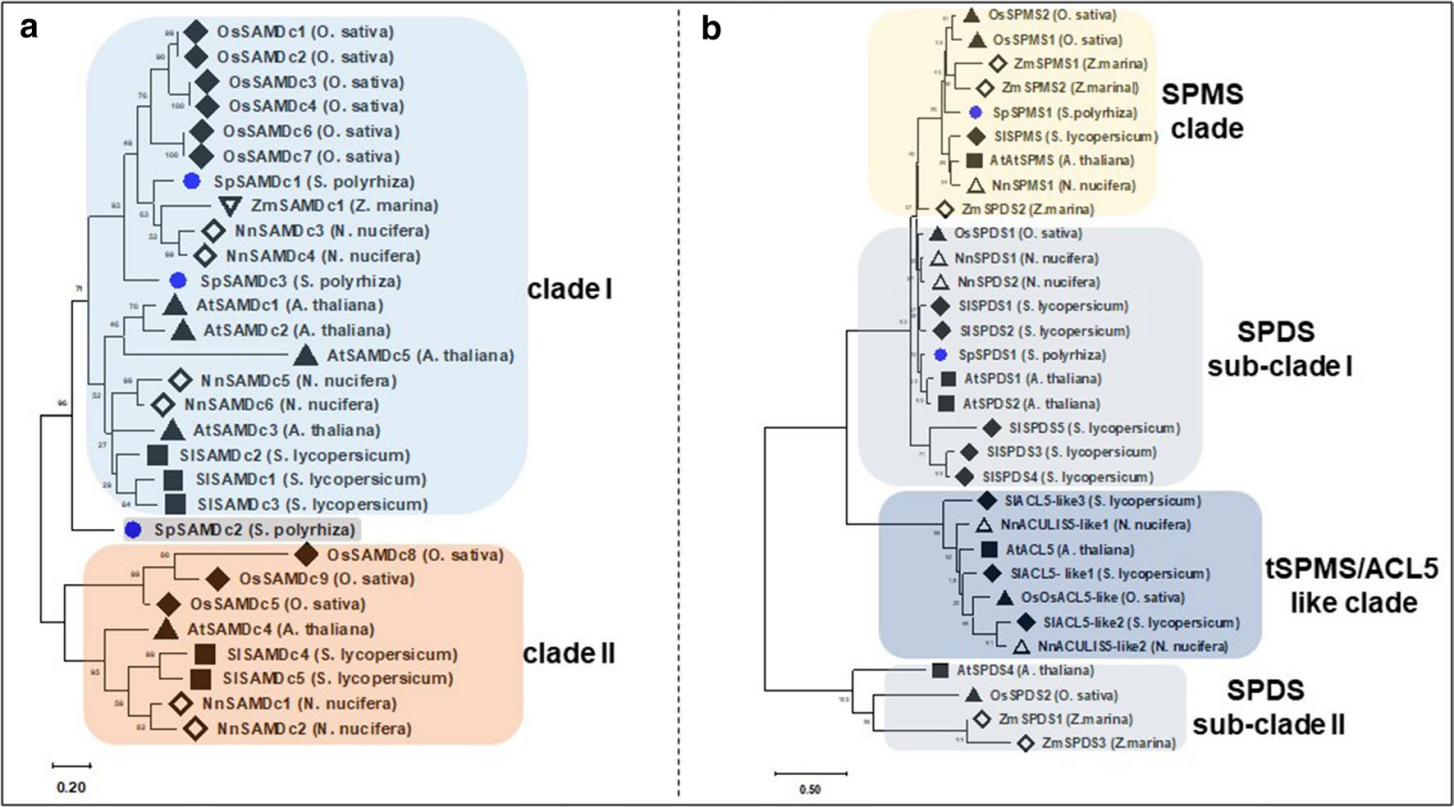
Molecular phylogenetic relatedness of duckweed *S-adenosylmethionine decarboxylases* and *spermidine synthases* genes in comparison to other plant gene species. Molecular phylogenetic relationships were derived from their evolutionary relationship based on the protein sequences from three land plants—*Arabidopsis*, tomato, rice and three aquatic plants (seagrass, lotus and duckweed). **a** Analysis of *S-adenosylmethionine decarboxylase (SAMDc)* involved 29 protein sequences from different plants. The SAMDc sequences were divided into two clades, although duckweed *SpSAMDc2* was not part of any clade. **b** Analysis of the *spermidine synthases (SPDS), spermine synthases (SPMS)* and *thermo-spermine synthases *(*tSPMS/ACULIS5*) involved 31 protein sequences. All sequences formed distinct clades for SPDS, tSPMS and SPMS. The evolutionary history was inferred using the Maximum Likelihood method based on the JTT matrix-based model. The bootstrap consensus tree inferred from 1000 replicates was taken to represent the evolutionary history of the taxa analyzed (Felsenstein 1985). All positions with less than 95% site coverage were eliminated. Evolutionary analyses were conducted in MEGA7 (Kumar et al. 2016). The bootstrap values of the confidence levels are shown as percentages at branch nodes

Next, phylogenetic reconstruction was carried out for SAMDc sequences (Fig. 4a). *SpSAMDc1* and *SpSAMDc3* were closely similar as compared to *SpSAMDc2*. In the reconstruction of SAMDc sequence phylogeny, *SpSAMDc1* and *SpSAMDc3* turned out to be a part of clade I, whereas *SpSAMDc2* did not fall into either clade I or clade II. These phylogenetic reconstructions indicate that *SpSAMDc3* is likely of a different origin than previously known SAMDc proteins from other plants, namely, tomato, rice and *Arabidopsis* (Fig. 4a).

Furthermore, a phylogenetic tree was constructed from pooled sequences for spermidine/spermine synthases (SPDS/SPMS) of various plants. For a broader overview, other aquatic plant seagrass and lotus sequences were also included along with land plant sequences (*Arabidopsis*, tomato, and rice). The protein sequences of SPDS, SPMS and thermospermine synthases (tSPMS/ACL5-like) were also included. All protein sequences fell into 4 distinct clades, two clades comprised of SPDS-like sequences, one clade comprised of SPMS-like sequences and another clade comprised of tSPMS/ACL5-like sequences (Fig. 4b). Duckweed *SpSPDS1* segregated with Arabidopsis and tomato SPDS-like sequences as a member of SPDS subclade I, while duckweed *SpSPMS1* fell into SPMS clade being closely related to SPMS sequences of tomato and seagrass. The fact that *SpSPMS1* fell into SPMS clade strengthens our annotation of this sequence as spermine synthase and not as spermidine synthase. The tSPMS/ACL5-like gene was not found present in the duckweed genome, and this clade contains protein sequences from other species and not duckweed.

### *S. polyrhiza* gene transcript abundance of PA biosynthesis pathway relative to ‘culture’ age

**Fig. 5:**
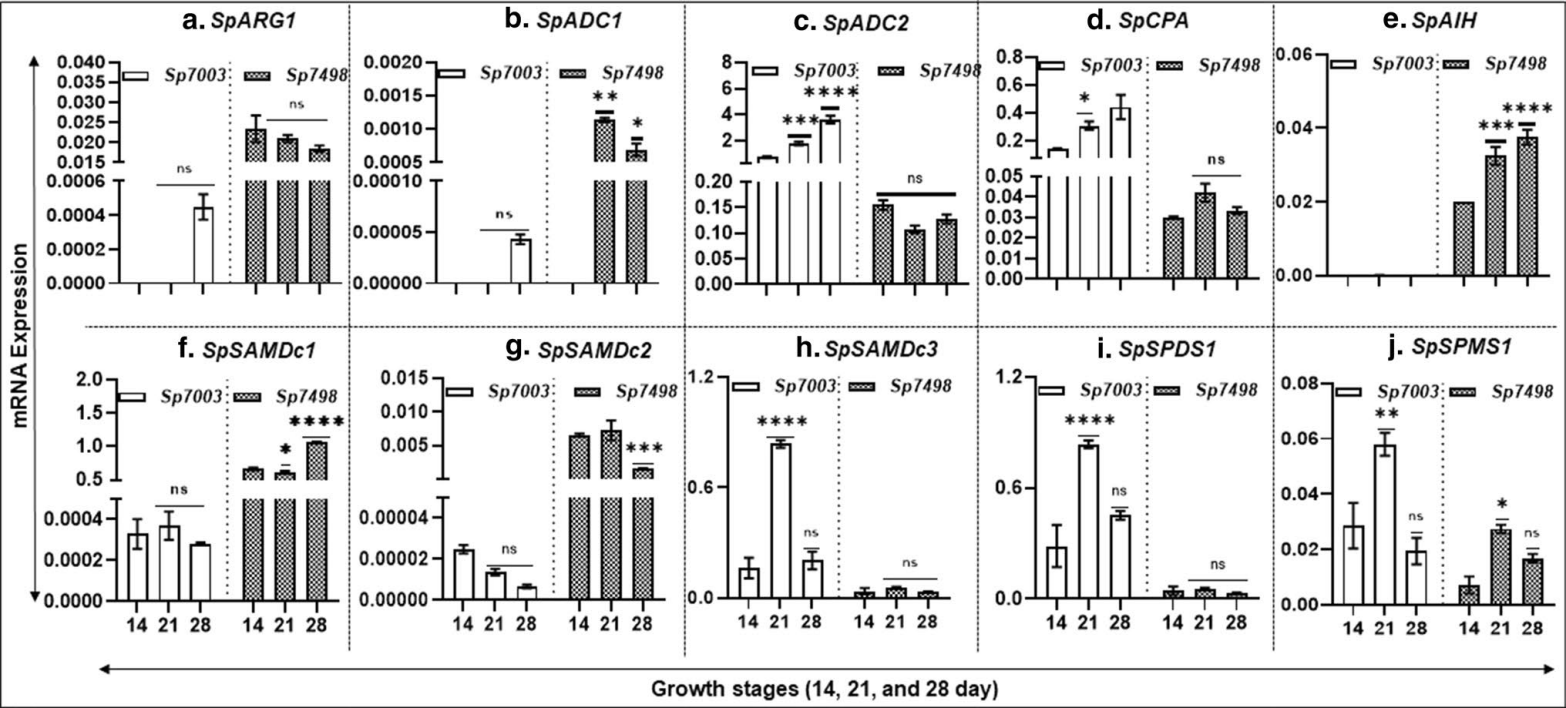
qRT-PCR analysis of PA biosynthetic pathway genes in two clones of *Spirodela polyrhiza*, Sp7498and Sp7003. Cultures were grown for 28 days in nutrient solution and samples were collected at days 14, 21 and 28 of growth. The confluence of plants in the culture flasks was seen on day 21of growth. mRNA expression profiles of whole plants from the two clones are shown, where *SpACT* and *Sp18SrRNA* housekeeping genes were used to normalize gene expression as described in the Materials and methods section. ANOVA with Dunnett's multiple comparisons test was performed for significant differences in gene expression in the aging plants. Statistical significance between expression data points were assessed against the day 14 expression profiles and categorized at *P < 0.05, **P < 0.01, and ***P < 0.001 using Graph Pad (version 8.0)

To validate whether or not *S. polyrhiza* genes are expressed, we characterized their transcript abundances in two clones of *S. polyrhiza,* Sp7498 and Sp7003. Both *S. polyrhiza,* Sp7498 and Sp7003 strains were grown as described in the methods section and harvested at days 14, 21 and 28. Changes in the gene transcript abundance of PA biosynthetic pathway relative to culture age were determined. It was apparent that PA gene transcript abundance was highly variable between two clones and, to some extent, also dependent upon the growth stages within a clone (Fig. 5). *SpARG1* was expressed only at day 28 in Sp7003, while in the Sp7498 strain, it was expressed at all the three growth stages at more or less similar levels (Fig. 5a). Similarly, *SpADC1* expression in Sp7003 was observed only at day 28, while in Sp7498 strain, it was expressed earlier, at days 21 and 28 (Fig. 5b). In comparison, *SpADC2* expression was higher in Sp7003 strain first observed at day 14, increasing thereafter at day 21 and further at day 28 (Fig. 5c). However, *SpADC2* expression in the Sp7498 strain remained the same at days 14, 21 and 28 but at a much lower level as compared to Sp7003 strain (Fig. 5c). The expression pattern of *SpCPA* gene was more or less similar to that seen for *SpADC2* in both Sp7003 and Sp7498 strains (Fig. 5d). Expression of *SpAIH* gene in Sp7003 was below the limit of detection but in the Sp7498 strain its expression increased from days 14 to 28 culture age (Fig. 5e).

Transcript abundance as a function of culture age was also determined for higher PA biosynthetic genes, such as *SpSAMDc1-3* and *SpSPDS1-2*. Expression of *SpSAMDc1,2* in Sp7003 and Sp7498 clones indicated measurable expression on day 14, remaining more or less steady or insignificant until day 28 in Sp7003 clone; however, more abundance of *SpSAMDc1,2* was apparent in Sp7498 clone from days 14 to 28 (Fig. 5f, g). In contrast, the expression patterns of *SpSAMDc3*, *SpSPDS1* and *SpSPMS1* were higher in Sp7003 clone than in the Sp7498 clone (Fig. 5h, i, j). Expression of both *SpSAMDc3* and *SpSPDS1* genes was negligible in Sp7498 clone. In contrast, *SpSPMS1* gene was expressed to a significant level in both clones at day 21 timepoint (Fig. 5j).

### Patterns of gene transcript abundance of PA biosynthesis pathway in response to exogenous MeJA

**Fig. 6:**
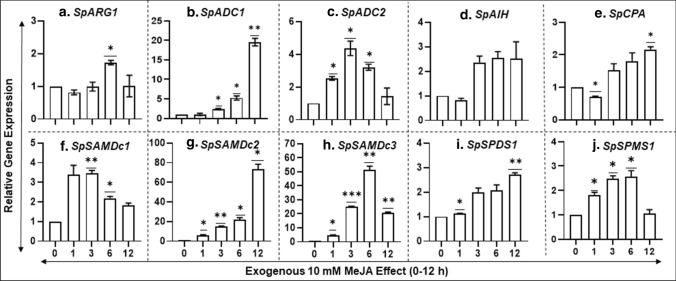
Effect of methyl jasmonate on the expression of PA biosynthesis genes in *S. polyrhiza***.** MeJA (10 μM) was added to 14-day-old *S. polyrhiza 7498* cultures as previously described (Upadhyay et al. 2020a, b). Samples were collected at 0, 1, 3, 6 and 12 h after treatment. Gene expression data were analyzed in treated and untreated fronds by qRT-PCR. Statistical significance between treatment data points was assessed with respect to control for each timepoint and categorized as *P < 0.05, **P < 0.01, and ***P < 0.0001 using graph pad (version 8.0)

Fourteen-day-old *S. polyrhiza* fronds were treated with 10 mM MeJA and sampled at 0, 1, 3, 6 and 12 h of treatment to determine the effects on the expression of different PA genes (Fig. 6). Putrescine (PUT) synthesis pathway gene *SpARG1* showed an expression spike only at 6 h of exposure to the hormone (Fig. 6a). On the other hand, among the two homologous arginine decarboxylase genes, S*pADC1* and *SpADC2*, highest expression of *SpADC1* was seen at 12 h and that of *SpADC2* maximized at 3 h and decreased thereafter (Fig. 6b, c). High expression of *SpAIH* gene was high at 3 h and remained so until 12 h, while *SpCPA* gene expression followed a similar trend as that seen above for *SpADC1* expression (Fig. 6d, e). All the tested PA biosynthetic genes, *SpSAMDc1-3*, *SpSPDS1* and *SpSPMS1*, were highly expressed in response to MeJA but each of them had a specific pattern (Fig. 6f–j).

### Gene transcript abundance of PA biosynthesis pathway in response to salinity

**Fig. 7:**
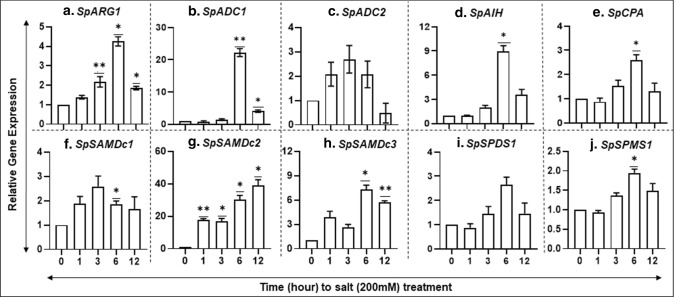
Comparative qRT-PCR analysis of genes of PA biosynthesis pathway in *S. polyrhiza *in response to salinity. Five fronds of *S. polyrhiza* 7498 were grown for 14 days, then 200 mM NaCl solution was added and samples were harvested at 0, 1, 3, 6 and 12 h (Upadhyay et al. 2019). Gene expression data were analyzed by qRT-PCR. The experiment was repeated three times (*n* = 3) with each sample size with approximately 100 fronds. ANOVA with Dunnett's multiple comparisons test was performed for significant differences. Statistical significance between data points was assessed against 0 h versus other timepoints of expression profiles using graph pad (version 8.0)

Salinity-induced (at 200 mM) expression of *SpARG1* (A)*, SpADC1* (B)*, SpAIH* (D), and *SpCPA* (E) genes was found highest in each case at 6 h of exposure (Fig. 7a–e). Like the responses to MeJA seen above (Fig. 6), *SpSAMDc1-3* and *SpSPMS1* genes were also highly expressed in response to salinity (Fig. 7f–h, j). These results indicate that *S. polyrhiza* responds to high salinity stress by upregulating genes in PA metabolic pathway (Fig. 7). A publicly available transcriptome data (Fu et al. 2019) for salinity-treated duckweeds was also analyzed and found to be enriched in only two genes, namely, *SpCPA* and *SpSAMDc2* (Supplementary Fig. 1).

### Acidic pH induces gene expression of PA biosynthesis pathway

**Fig. 8:**
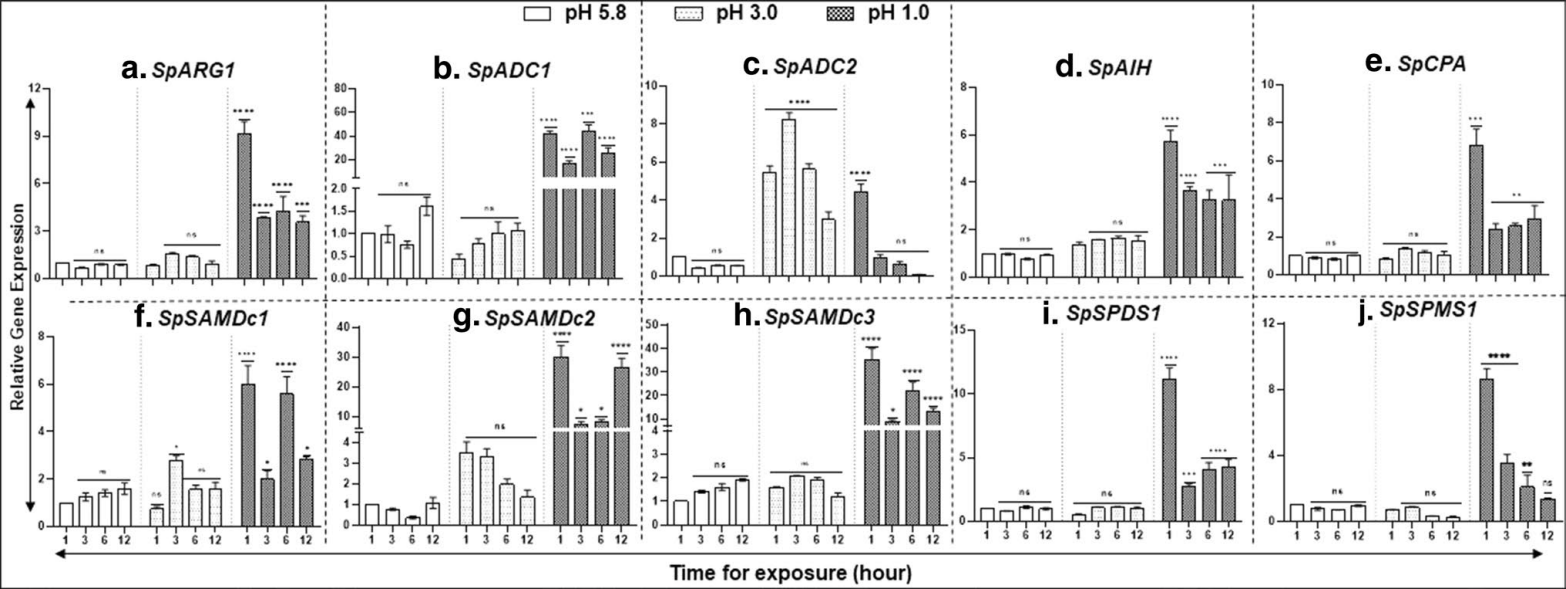
Comparative qRT-PCR analysis of genes of PA biosynthesis pathway in *S. polyrhiza *under acidic pH conditions. Five fronds of *S. polyrhiza* 7498 were grown for 14 days. 100 fronds were placed in three pH conditions (1.0, 3.0, 5.8) then samples were harvested at 0, 1, 3, 6 and 12 h. Gene expression data were analyzed by qRT-PCR. The experiment was repeated three times (*n* = 3) with each sample size with approximately 100 fronds. ANOVA with Dunnett's multiple comparisons test was performed for significant differences. Statistical significance between data points was assessed against pH 5.8 (control) timepoints versus other timepoints (pH 1.0 and pH 3.0) of expression profiles using graph pad (version 8.0)

To discern the effect of acidic pH on PA pathway genes,14-day-old *S. polyrhiza* plants were separately grown for 12 h at three different pH conditions: pH 5.8 (control), pH 3.0, and pH 1.0. Gene expression analysis revealed that all the 10 genes including *SpARG1; SpADC1, 2; SpAIH;* and *SpCPA* (Fig. 8a–e), three SAMDc genes (Fig. 8f–h), *SpSPDS1* (Fig. 8i) and *SpSPMS1* (Fig. 8j) increased in expression at pH 1.0 to a high degree, while only *SpADC2* was significantly expressed at pH 3.0. Such a differential gene expression was not observed at pH 5.8.

### Temperature stress mediated transcriptional modulation of PA biosynthesis pathway

**Fig. 9:**
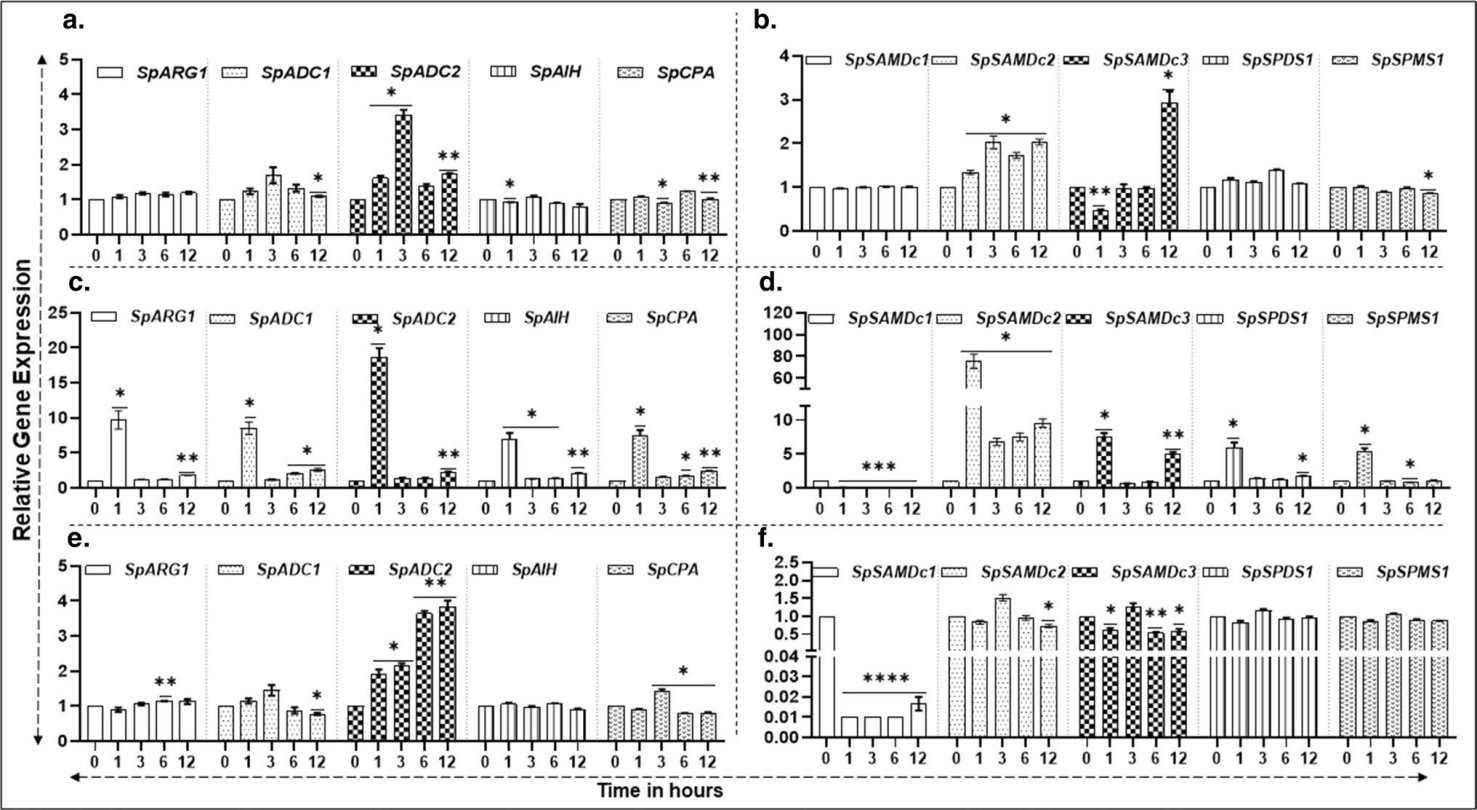
Day time and temperature stress mediated modulation of PA biosynthetic pathway in duckweed (*S. polyrhiza L.*). Time of day/control conditions sampling (8.00 am–8.00 pm EST) along with heat (42 °C temperature) and cold (4 °C temperature) treatments included time 0, 1, 3, 6, 12 h. Total RNA was isolated from these samples and cDNA was made as described in materials and method section. Day time had impact on gene expression of **a** *SpADC2* and **b** *SpSAMDc2* and *SpSAMDc3* was larger although minor but significant changes were seen in some genes. Most of the genes involved in **c** PUT biosynthesis and **d** SPD and SPM biosynthesis were transiently induced (> twofold) upon heat exposure at 1 h. Cold exposure had much lower impact on most of the genes involved in **e** PUT biosynthesis and **f** SPD and SPM biosynthesis except for *SpADC2* and *SpSAMDc1*. Gene expression data were analyzed by qRT-PCR. The experiment was repeated three times (*n* = 3) with each sample size of approximately 100 fronds. ANOVA with Dunnett’s multiple comparisons test was performed for significant differences. Statistical significance between data points was assessed against 0 h (control) timepoints versus other timepoints of expression profiles using graph pad (version 8.0) and categorized at *P < 0.05, **P < 0.01, and ***P < 0.001 using Graph Pad (version 8.0)

Temperature stress treatments involved heat (42 °C) and cold (4 °C). Expression was assessed at 0, 1, 3, 6, 12 h timepoints for 10 genes of PA biosynthetic pathway. To eliminate a possibility of variation of genes during day time, a control was run along with similar timepoints. In control conditions (without treatment), except for *SpADC2*, *SpSAMDc2* and *SpSAMDc3*, the rest of the seven other genes were expressed unchanged throughout the day length (8am–8 pm EST) (Fig. 9a, b). In response to heat, an early transient response was observed in the expression of 9 genes except for *SpSAMDc1*. The expression of PUT biosynthesis pathway genes *SpARG1*, *SpADC1*, 2, *SpAIH,* and *SpCPA* increased at 1 h, with a decline thereafter till 12 h. Similar patterns were observed for *SpSAMDc3, SpSPDS1* and *SpSPMS1* genes, while *SpSAMDc1* expression was down regulated. Notably, *SpSAMDc2* expression was upregulated at all stages in response to heat stress (Fig. 9c). Contrary to heat stress, cold stress increased the expression of PA biosynthesis gene *SpADC2* at 6 h and 12 h which significantly peaked at 12 h (Fig. 9e, f). A significant decline of *SpSAMDc1* gene was observed starting from 1 h and continued until 12 h (Fig. 9f). Rest of the 8 genes were not affected by cold stress (Fig. 9e, f).

## Discussion

**Fig. 10:**
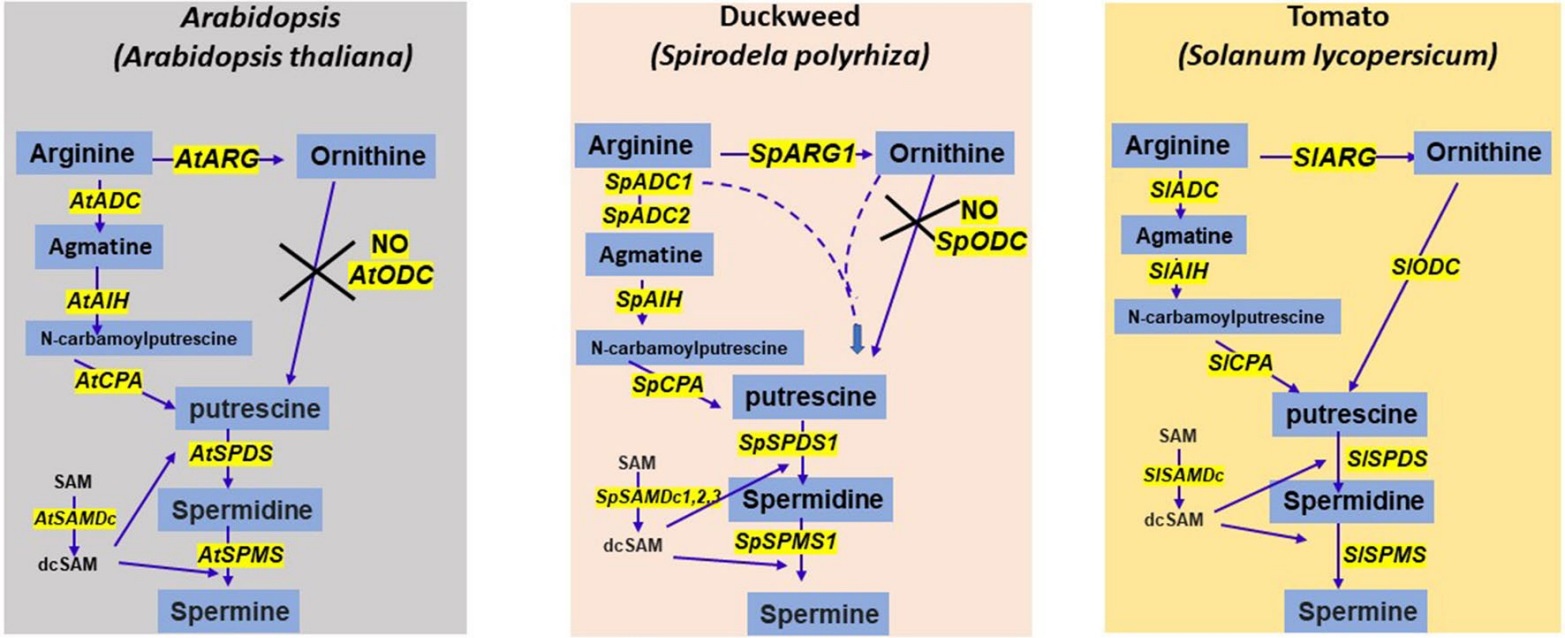
Schematic representation of PA biosynthetic pathway in duckweed, tomato and *Arabidopsis*. Comparative analysis of PA biosynthetic pathway in duckweed versus land plants shows the absence of ODC pathway in duckweed as in *Arabidopsis,* while tomato harbors both ADC and ODC pathways. Like the bacterial ADC gene AdiA (Tolbert et al. 2003; Andréll et al. 2009; Deka et al. 2017) and *Lactobacillus ODC gene* (*L30a OrnDC*) (Momany et al. 1995), one of the duckweed ADC was found to be of prokaryotic origin which may utilize multiple substrates, such as lysine and ornithine

Duckweed (Lemnaceae) family with highly reduced anatomies (Hillman 1961, 1976) has emerged as a model of aquatic higher plants for high protein value and biomanufacturing (Rusoff et al. 1980; Stomp 2005; Wang et al. 2014; Van Hoeck et al. 2015; Appenroth et al. 2015; Edelman and Colt 2016). In addition, they have been valuable in studies on photosynthesis, photosystem II in particular (Mattoo et al. 1989; Edelman and Mattoo 2005, 2008) as well as for ecotoxicological studies and phytoremediation (Ziegler et al. 2016). Polyamines (PAs) as aliphatic biogenic amines have been shown to be critical for various aspects of plant growth, development, and other cellular processes in higher plants (see “Introduction”). Here, we have identified and characterized 10 genes in PA biosynthetic pathway in duckweed, *Spirodela polyrhiza* L, together with a comparative assessment of these genes to those known in Arabidopsis and tomato (Fig. 10).

In comparison to the two arginases encoded in *Arabidopsis* and tomato genomes (Liu et al. 2018; Upadhyay et al. 2020b), only a single arginase gene (*SpARG1*) was found in the duckweed genome. Similar to Arabidopsis, tomato and rice, two arginine decarboxylases (ADCs) are also present in the duckweed genome (*S. polyrhiza*) (Tables 1, 3). Together with these, the presence of single *SpAIH* and *SpCPA* genes strengthens a functional ADC type pathway of polyamine biosynthesis in duckweed (Table 1). Moreover, both duckweed ADCs are related to two different clades of amino acid decarboxylases. Both ADC clades distinctly segregate as either of bacterial or cyanobacterial origin. Interestingly, each group also contains *E. coli* K12-like two ADC genes, *ADIA* and *SPEA,* which are known as biosynthetic and biodegradative/catabolic type of arginine decarboxylases, respectively (Tabor and Tabor 1985). Based on the presence of bacterial-type ADCs in two distinct clades, these were named as group III biosynthetic type ADC clade and group IV biodegradative type ADC clade. None of the known ADCs from Arabidopsis, tomato and rice were part of the latter group. In addition, *SpADC1* as part of a clade similar to cyanobacteria raises the possibility of their similar evolutionary origin.

Most of the known characterized plant ADCs in Arabidopsisi, tomato and rice belong to group IV (biosynthetic type) and none were identified from group III (biodegradative type). Group III ornithine/lysine/arginine decarboxylases are of bacterial origin (Sandmeier et al. 1994) which strengthens segregation of *SpADC1* along with biodegradative clade in phylogeny. It is also evident that group III amino acid decarboxylases *Lactobacillus 30a* bacterial ODC gene (*L30a OrnDC*) can utilize multiple substrates, such as ornithine, arginine and lysine (Momany et al. 1995). The fact that in our studies *SpADC1* also fell in the same clade raises the possibility that duckweed *SpADC1* may also utilize multiple substrates but these suggestions need to be further explored. It is noted here that there was only 24% sequence similarity at the amino acid level between the SpADC1 and *Lactobacillus* L30aOrnDC which is low for deciphering any functional analogy. The known land plant ADCs in *Arabidopsis*, rice and tomato do not have this pfam profile, and therefore, they do not fall into this group. The presence of pfam motif in *SpADC1* suggests that it is a prokaryotic/biodegradative type ADC gene, while *SpADC2* is a typical land plant type, since the latter possesses domains such as the ADCs sequences from *Arabidopsis*, tomato and rice (Fig. 2). Such ADC sequences are also present in lotus (*N. nucifera*) and some land plants.

ODC pathway is another route for putrescine synthesis; however, this pathway seems to be absent in *S. polyrhiza*. It is noted here that neither in the released assembly (Sp7498v2) at phytozome nor our analysis of tomato and rice ODC query sequences against new Sp7498v3 and Sp9509v2 genome assemblies yielded any ODC-like sequence. Thus, PA pathway in the duckweed *S. polyrhiza* in terms of a functional ODC gene resembles *Arabidopsis* in which ODC gene has thus far not been found (Hanfrey et al. 2001). The aquatic lotus (*N. nucifera*) has one locus for complete *NnODC* gene. The fact that three *bona fide* genetic loci in *S. polyrhiza* for S-adenosylmethionine decarboxylase and one locus each for spermidine synthase and spermine synthase were found in the present investigation suggest the presence of complete pathway for the synthesis of SPD and SPM.

In the open water bodies, bacterial and fungal infections are very prevalent. The manner in which aquatic plants manage defense against such deterants is still fragmentary at best. Lipoxygenase pathway vis a vis the presence of hormone methyl jasmonic acid (JA/MeJA) is known in duckweed (Upadhyay et al. 2020a). MeJA was found to upregulate transcription of a majority of the PA pathway genes. The nature of MeJA-induced responses vis a vis PAs needs to be ascertained.

Duckweed are known to possess phytoremediation properties, since aquatic life is prone to challenges by severe contamination in water bodies including salinity. Since salinity was found to induce genes of the PA metabolic pathway in the duckweed suggest that PA pathway likely provides protection to duckweed. In this context, it is noted that acl5/Spms *Arabidopsis* mutant which lacks Spm was found to be hypersensitive to salinity and drought (Yamaguchi et al. 2007).

Acid induced arginine decarboxylase (AdiA) forms a part of an enzymatic system in *E. coli*, *S. typhimurium* and methane-producing *M. jannaschii* which makes these organisms acid-resistant and lets them survive in a highly acidic medium (Tolbert et al. 2003; Andréll et al. 2009; Deka et al. 2017). The finding presented in our study that duckweed *SpADC1* possesses a bacterial biodegradative ADC type HMM profile and the fact that all the 10 genes tested responded to pH 1.0 to a high degree indicate involvement of *SpADC1* in the catabolic arginine pathway as has been found in *E. coli* strain K12 (Tabor and Tabor 1985). Higher expression of both the duckweed ADCs presented in this study is suggestive of an anabolic type of arginine pathway for putrescine synthesis at low pH conditions.

Time of day and temperature expreme conditions influence PA biosynthetic pathway at genetic level in land plants, such as tomato (Upadhyay et al. 2020a, b). Temperature stress effects on PA biosynthesis pathway in aquatic plants such as duckweed (*S. polyrhiza*) are not known. We found that the day length has minimal effect on PA biosynthetic pathway genes except for changes in the expression of *SpADC2, SpSAMDc2* and *SpSAMDc3* genes. On the other hand, exposure to heat transiently induced a majority of PA biosynthetic pathway genes, while least impact on PA genes was observed under cold stress in duckweed (*S. polyrhiza*) (Fig. 9).

ADC pathway but not ODC pathway has been reported in Arabidopsis based on the presence of cognate genes (Hanfrey et al. 2001). In tomato and tobacco model plants, genes encoding for ADC and ODC pathways have been identified (Liu, et al. 2018; Upadhyay et al. 2020a,b; Hidalgo et al. 2020). In light of the published data, a schematic comparison of known components of PA biosynthetic pathway (based on identified genes) in *Spirodela* are presented in Fig. 10. Even though no gene sequence was found encoding for ornithine decarboxylase (ODC) it is possible that *SpADC1* could also work as an ODC-like gene due to presence of HMM signature motifs for group III ADCs (biodegradative type ADCs) and it might also utilize ornithine as alternate substrate along with arginine. This would result in an operational ODC pathway for PA synthesis even in the absence of a direct encoding sequence for ODC enzyme. However, this possibility needs to be experimentally validated. It is noted here that ODC activity in *Lemna* species has been shown (Adomas et al. 2020). However, in the latter investigation, the assessment for ODC and ADC activities was based solely on the release of CO_2_ which is subject to further assessment via more deeper studies. Until each gene is individually cloned and thereafter tested for enzyme activities the presence or absence of ODC pathway in duckweed is doughtful. Our studies presented here are novel for the identification/characterization of each genetic component based on available genome assemblies for *Spirodela* (*S. polyrhiza*) species in relation to polyamine biology in this important aquatic plant species.

### *Author contribution statement*

Conceived and designed the study: RKU; Computational Bioinformatics: RKU; Gene modeling: RKU, JS; Performed the experiments: RKU; Analyzed the data: RKU; Original draft: RKU; Finalized the manuscript: AKM. Funding and reagent availability: AKM; All authors approved the final version.

## Supplementary Information

Below is the link to the electronic supplementary material.

Supplementary file1 (DOCX 48 KB)

## Abbreviations

PAs: Polyamines
ADC: Arginine decarboxylase
ODC: Ornithine decarboxylase
HMM: Hidden Markov model
qRT-PCR: Quantitative real-time polymerase chain reaction
MeJA: Methyl jasmonate
