# Supplementary material for: Genomic analysis of the polyamine biosynthesis pathway in duckweed Spirodela polyrhiza L.: presence of the arginine decarboxylase pathway, absence of the ornithine decarboxylase pathway, and response to abiotic stresses
Source: Planta. 2021 Oct 25;254(5):108. doi: 10.1007/s00425-021-03755-5 (PMC8545783; doi:10.1007/s00425-021-03755-5)

**Supplementary Information**

**Genomic analysis of the polyamine (PA) biosynthesis pathway in the duckweed *Spirodela polyrhiza* L.:  presence of the arginine decarboxylase pathway, absence of the ornithine decarboxylase encoding genes, and response to stress conditions**

**Rakesh K. Upadhyay^1*^, Jonathan Shao^2^ and Autar K. Mattoo^1*^**

**^1^**Sustainable Agricultural Systems Laboratory, United States Department of Agriculture, Agricultural Research Service, Henry A. Wallace Beltsville Agricultural Research Center, Beltsville, MD 20705-2350, USA. **^2^**Bioinformatics- North East Area Office, United States Department of Agriculture, Agricultural Research Service, Henry A. Wallace Beltsville Agricultural Research Center, Beltsville, MD 20705-2350, USA.

*Authors for correspondences:

RKU ([rakesh.upadhyay@usda.gov](mailto:rakesh.upadhyay@usda.gov)); AKM ([autar.mattoo@usda.gov](mailto:autar.mattoo@usda.gov))

ORCID iD: RKU (0000-0001-6742-5853); SJ (0000-0002-0862-8172); AKM (0000-0001-9226-7164)

**Supplementary Information**

**Supplementary Table 1:** Protein sequence accession numbers used to generate ADC/ODC/DAPDC phylogenetic trees.

**Supplementary Table 2:** Protein sequence accession numbers used to generate SAMDc phylogenetic trees.

**Supplementary Table 3:** Protein sequence accession numbers used to generate SPDS/SPMS/ACL5-like phylogenetic trees.

**Supplementary Table 4:** Primer sequences for polyamine biosynthetic pathway genes used for qRT-PCR analysis

**Supplementary Figure 1:** Two enriched genes from Duckweed PA pathway are differentially regulated in salt induced transcriptome.

**Supplementary Table 1: Protein sequence accession numbers used to generate ADC/ODC/DAPDC phylogenetic trees.**

| Duckweed  (*S. polyrhiza*) | SpADC1 (Spipo9G0068800), SpADC2 (Spipo16G0022600), SpDapDc1 (Spipo8G0054200), |
| --- | --- |
| Tomato  (*S. lycopersicum*) | SlADC1(Solyc01g110440), SlADC2(Solyc10g054440), SlODC1 (Solyc04g082030), SlODC2(Solyc03g098300), SlODC3 (Solyc03g098310) |
| Arabidopsis  (*A. thaliana*) | AtADC2(AT4G34710), AtADC1(AT2G16500), AtDapDC1(AT3G14390), AtDapDC2(AT5G11880) |
| *Rice* (*O. sativa*) | OsADC1(Os04g01690), OsADC2 (Os06g04070), OsODC1 (Os02g28110), OsODC2 (Os04g04980), OsODC3 (Os09g37120), OsDapDC1(Os02g24354), |
| Lotus  (*N. nucifera*) | NnADC1 (XP_010271459), NnADC2 (XP_010263903), NnADC3 (XP_010245952), NnODC1 (XP_010266064), NnDapDc1 (XP_019053047), NnDapDc2 (XP_010263591) |
| *Eelgrass*  *(Z. marina)* | ZmADC1 (Zosma1g02550), ZmDapDC1(Zosma234g00330), |
| Protein sequences from NCBI blastp with SpADC1 and SpADC2 as query | LjODC1 (*L. japonicus*\|CAE02644.1), LcL/ODC1(L. clavatum\|BAR42911), PdADC (*P. dactylifera*\|XP_017699151.2), EgADC (*E. guineensis*\|XP_010927667.1), DcADC (*D. cayenensis*\|XP_039139138.1), PdADC (*P. dulcis*\|XP_034202124.1), PaADC (*P. alba*\|XP_034906233.1), JrADC (*J. regia*\|XP_035546814.1), AoADC (*A. officinalis*\|XP_020261771.1), DcADC1 (*D. cayenensis*\|XP_039139139.1), CeADC (*C. esculenta*\|MQM12524.1), DcADC *(**D. catenatum*\|XP_028552740.1), PeADC (*P. equestris*\|XP_020582701.1), AsADC (*A. shenzhenica*\|PKA58021.1), CnADC (*C. nucifera*\|KAG1360570.1), ClADC (*C. littledalei*\|KAF3332346.1), AcADC (*A. comosus*\|XP_020114907.1), OeADC (*O. europaea*\|XP_022869017.1), SPEA (*E. coli* K12\|P21170), ADIA (*E. coli* K12\|P28629), ADC (*Fischerella*\|WP_026086470.1), ADC (*Nostoc*\|RCJ37089.1), ODC (*Lactobacillus30A*\|P43099.2), ADC-like (*Planktothrix*\|HBK22293.1), ADC (*Geitlerinema*\|WP_015173045.1), ADC (*Oscillatoriales*\|OIP69442.1) |
| *Archaea* | SPEA (*E. coli* K12\|P21170), ADIA (*E. coli* K12\|P28629), ADC (*Fischerella*\|WP_026086470.1), ADC (*Nostoc*\|RCJ37089.1), ODC (*Lactobacillus30A*\|P43099.2), ADC-like (*Planktothrix*\|HBK22293.1), ADC (*Geitlerinema*\|WP_015173045.1), ADC (*Oscillatoriales*\|OIP69442.1) |

**Supplementary Table 2: Protein sequence accession numbers used to generate SAMDc phylogenetic trees.**

| Duckweed  (*S. polyrhiza*) | SpSAMDc1 (Spipo1G0013800), SpSAMDc2 (Spipo5G0023000), SpSAMDc3 (Spipo9G0049500) |
| --- | --- |
| Tomato  (*S. lycopersicum*) | SlSAMDc1 (Solyc05g010420.1.1), SlSAMDc2 (Solyc02g089610.1.1), SlSAMDc3 (Solyc01g010050.2.1), SlSAMDc4 (Solyc01g080380.2.1), SlSAMDc5 (Solyc06g054460.1.1) |
| Arabidopsis  (*A. thaliana*) | AtSAMDc1(At3G02470.1), AtSAMDc2 (At5G15950.1), AtSAMDc3 (At3G25570.1), AtSAMDc4 (At5G18930.1), AtSAMDc5 (At3G17715) |
| *Rice* (*O. sativa*) | OsSAMDc1 (Os02g39790.2), OsSAMDc2 (Os02g39795.1), OsSAMDc3 (Os04g42090.3), OsSAMDc4 (Os04g42095.1), OsSAMDc5 (Os05g04990.1), OsSAMDc6 (Os09g25620.1), OsSAMDc7 (Os09g25625.1), OsSAMDc8 (Os05g13480.1), OsSAMDc9 (Os09g24600.1), |
| Lotus  (*N. nucifera*) | NnSAMDc1 (XP_010279141.1), NnSAMDc3 (XP_010268432.1), NnSAMDc2 (XP_010255822.1), NnSAMDc4 (XP_010277985.2), NnSAMDc5 (XP_010261810.2), NnSAMDc6 (XP_019053457.1), |
| *Eelgrass*  *(Z. marina)* | ZmSAMDc1 (Zosma35g00770.1) |

**Supplementary Table 3: Protein sequence accession numbers used to generate SPDS/SPMS/ACL5-like phylogenetic trees.**

| Duckweed  (*S. polyrhiza*) | SpSPDS1 (Spipo12G0011300), SpSPMS1(Spipo26G0016400) |
| --- | --- |
| Tomato  (*S. lycopersicum*) | SlSPDS1 (Solyc04g026030.2.1), SlSPDS2 (Solyc05g005710.2.1)  SlSPDS3 (Solyc08g014310.2.1), SlSPDS4 (Solyc06g053510.2.1)  SlSPDS5 (Solyc06g053520.2.1), SlACL5- like1 (Solyc08g061970.2.1)  SlACL5-like2 (Solyc09g075900.2.1), SlACL5-like3 (Solyc07g041300.1.1)  SlSPMS (Solyc03g007240.2.1) |
| Arabidopsis  (*A. thaliana*) | AtSPDS1 (At1G23820), AtSPDS2 (At1G70310), AtSPMS(At5G53120), AtACL5 (At5G19530.1), AtSPDS4 (At5G04610.1) |
| *Rice*  (*O. sativa*) | OsSPDS1 (Os07g22600.1), OsSPDS2 (Os08g40890.1), OsSPMS1 (Os06g33710.2), OsSPMS2 (Os02g15550.2), OsOsACL5-like (Os02g14190.1) |
| Lotus  (*N. nucifera*) | NnSPDS1 (XP_010271957.1), NnSPDS2 (XP_010251305.1)  NnSPMS1 (XP_010261580.1), NnACULIS5-like2 (P_010277477.1)  NnACULIS5-like1 (XP_010261941.1) |
| *Eelgrass*  *(Z. marina)* | ZmSPDS1 (Zosma27g01060.1), ZmSPDS2 (Zosma54g00880.1)  ZmSPDS3 (Zosma235g00120.1), ZmSPMS1 (Zosma2g03130.1)  ZmSPMS2 (Zosma166g00110.1) |

**Supplementary Table 4: Primer sequences for polyamine biosynthetic pathway genes used for qRT-PCR analysis**

| Gene Name | Sequence ID | Forward | Reverse |
| --- | --- | --- | --- |
| *Actin* | Spipo17G0011400 | GGCTACTCCTTCACCACCAC | GCTCGTAGGTCTTCTCGACG |
| *18S rRNA* | Spipo23G0000600 | CGGTCCTATTGTGTTGGCCT | TCCTTGGCAAATGCTTTCGC |
| SpARG1 | Spipo17G0020700 | GTCGACGTGGACTGTCTGGAT | CCACGTAGGTTGTGCAAGATGT |
| SpADC1 | Spipo9G0068800 | GAGGTTGAAGACGGCAATGG | CTTCCTCTTTCTGGCGAAGAAC |
| SpADC2 | Spipo16G0022600 | CCTTCCACTCCATGCCTTATCT | CGGAGGAGAACATGGAAGAGAG |
| SpCPA | Spipo0G0005000 | CATCACCTTTTATGGCAACTCG | CAGCTGTGCCTCTGAGATTTGA |
| SpAIH | Spipo25G0016900 | GACTCCCCGCCTCATACATAAA | TGGTATGCTTCATCATCCCATC |
| SpSPDS1 | Spipo12G0011300 | CTGTGAACTATGCCTGGACGAC | AGAGGGCGTCTGAAGTCAACTG |
| SpSPMS1 | Spipo26G0016400 | GCGTTCTCTGTAATCAGGCAGA | CCAATCGTGCCACTAGGATATG |
| SpSAMDc1 | Spipo1G0013800 | CTCTCTCAACCATCCACGTCAC | CAAAGCATCTCAGGACCCTCTC |
| SpSAMDc2 | Spipo5G0023000 | GAGGGAGCGCTCAAGTGTTT | GGCAAGGTACCCTGAAAAGCTC |
| SpSAMDc3 | Spipo9G0049500 | GCTCTCAGAGTCCAGCCTCTTC | GAATTCTTGGTATCGCCAGGAG |

**Supplementary Figure 1:** Two enriched genes from Duckweed PA pathway are differentially regulated in salt induced transcriptome.


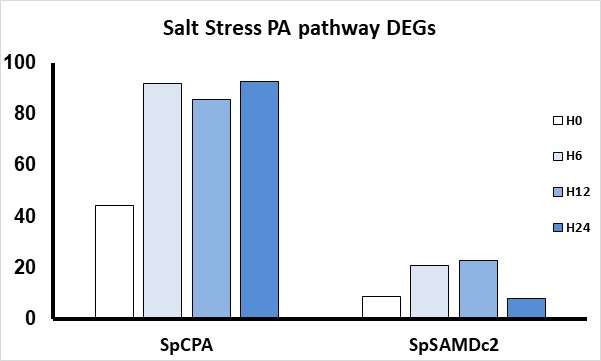

Supplement: Supplementary file 1 — Supplementary file1 (DOCX 48 KB) [file 425_2021_3755_MOESM1_ESM.docx]
